# Pain management of unicompartmental (UKA) vs. total knee arthroplasty (TKA) based on a matched pair analysis of 4144 cases

**DOI:** 10.1038/s41598-020-74986-x

**Published:** 2020-10-19

**Authors:** Franziska Leiss, Julia Sabrina Götz, Günther Maderbacher, Florian Zeman, Winfried Meissner, Joachim Grifka, Achim Benditz, Felix Greimel

**Affiliations:** 1 Department of Orthopedics, University Medical Center Regensburg, Asklepios Klinikum Bad Abbach, Kaiser-Karl-V.-Allee 3, 93077 Bad Abbach, Germany; 2 Center for Clinical Studies, University Medical Center of Regensburg, Franz-Josef-Strauss-Allee 11, 93053 Regensburg, Germany; 3 Department of Anesthesiology and Intensive Care, Jena University Hospital, Am Klinikum 1, 07747 Jena, Germany

**Keywords:** Medical research, Outcomes research, Orthopaedics

## Abstract

Unicompartmental knee arthroplasty and total knee arthroplasty are well established treatment options for end-stage osteoarthritis, UKA still remains infrequently used if you take all knee arthroplasties into account. An important factor following knee arthroplasty is pain control in the perioperative experience, as high postoperative pain level is associated with persistent postsurgical pain. There is little literature which describes pain values and the need for pain medication following UKA and/or TKA. So far, no significant difference in pain has been found between UKA and TKA. The aim of the study was to evaluate differences in the postoperative course in unicompartmental knee arthroplasty vs. total knee arthroplasty regarding the need for pain medication and patient-reported outcomes including pain scores and side effects. We hypothesized that unicompartmental knee arthroplasty is superior to total knee arthroplasty in terms of postoperative pain values and the need of pain medication. In this project, we evaluated 2117 patients who had unicompartmental knee arthroplasty and 3798 who had total knee arthroplasty performed, from 2015 to 2018. A total of 4144 patients could be compared after performing the matched pair analysis. A professional team was used for data collection and short patient interviews to achieve high data quality on the first postoperative day. Parameters were compared after performing a 1:1 matched pair analysis, multicenter-wide in 14 orthopedic departments. Pain scores were significantly lower for the UKA group than those of the TKA group (*p* < 0.001 respectively for activity pain, minimum and maximum pain). In the recovery unit, there was less need for pain medication in patients with UKA (*p* = 0.004 for non-opioids). The opiate consumption was similarly lower for the UKA group, but not statistically significant (*p* = 0.15). In the ward, the UKA group needed less opioids (*p* < 0.001). Patient subjective parameters were significantly better for UKA. After implantation of unicompartmental knee arthroplasty, patients showed lower pain scores, a reduced need for pain medication and better patient subjective parameters in the early postoperative course in this study.

## Introduction

If conservative treatment fails, total knee arthroplasty (TKA) and unicompartmental knee arthroplasty (UKA) are good and well-established treatment options for end-stage arthritis, while UKA is confined to a single compartment of the knee. The medial compartment is associated with a higher incidence of arthritis compared to the lateral compartment, therefore medial UKA is performed more often than lateral UKA. Because of the anatomic and kinematic differences between the medial and lateral compartment, lateral UKA is technically more challenging than medial UKA^1,2^.

There are controversial discussions, whether a retro-patellar replacement should be performed primarily for prosthesis implantation. A uniform recommendation has not yet been issued^3–5^.

UKA has been shown to have several advantages over TKA, including reduced blood loss, shorter length of hospitalization, improved postoperative patient-reported functional outcomes and less postoperative morbidity^6–10^. Unicompartmental knee arthroplasty allows patients a faster return to a more functional level than TKA, but postoperative pain management still remains a challenge, since there is no significant difference in pain^11–16^. Pain negatively affects the functional outcome, patient satisfaction and their psychological well-being^17–19^. Postoperative pain management strategies include oral or intravenous analgesics, patient-controlled analgesia (PCA), single shot or continuous peripheral nerve blocks or local infiltration analgesia (LIA). The aim of postoperative pain management is to improve the patients’ comfort, satisfaction and functional outcome after UKA and TKA. Insufficient pain management can be revealed by Continuous Quality Improvement (CQI) strategies. The “Quality Improvement in Postoperative Pain Management (QUIPS)” project is an outstanding tool^20^ to compare and then improve pain management. Despite the fact that the successful use of a knee arthroplasty increases the quality of the patient’s life, 20–30% of all patients remain permanently dissatisfied with the results of their operation^21^. Kehlet et al. reported, that 10–34% of the patients may develop chronic pain after implantation of TKA. A high postoperative pain level is associated with persistent postsurgical pain^22,23^. Previous studies have mainly used PROM scores (WOMAC/KSS) to assess postoperative pain in the follow-up after surgery. An investigation of early postoperative pain, the need of pain medication, side effects and functional impairments after UKA and/or TKA has not been considered extensively so far.

The purpose of this study was to evaluate differences in the short-term perioperative course after unicompartmental knee arthroplasty vs. total knee arthroplasty. We also looked at the need for pain medication and patient-reported outcomes as well as pain intensity and side effects, since total knee arthroplasty still is, by far, the more frequently used technique. In a relevant amount of cases, UKA could have been used considering indication criteria^24,25^.

This large-scale multicenter study evaluated the need for pain medication, subjective functional score, as well as pain intensity scores in the immediate postoperative course of unicompartmental or total knee arthroplasty. We assumed that unicompartmental knee arthroplasty is superior to total knee arthroplasty, in terms of postoperative pain and the need of pain medication.

## Material and methods

The QUIPS (“Quality Improvement in Postoperative Pain Management”) project is an initiative to compare patient-reported outcomes related to perioperative pain management. With over 450,000 data records, QUIPS is one of the largest acute pain databases in the world. In participating hospitals of the QUIPS project, data was obtained on the first postoperative day. The QUIPS project is supported by the German Society of Surgeons and the German Society of Anesthesiologists^20,26^. The study was approved by the Ethics Committee, as well as, the Data Security Board of the Jena University Hospital, Jena, Germany, and by the Ethics Committee of the University of Regensburg. Furthermore, the project was registered in the German Register of Clinical Studies (DRKS) with the approval number DRKS00006153 (WHO register). The study was applied in accordance with the ethical standards of the Declaration of Helsinki 1975.

All primary unicompartmental and total knee arthroplasties included in the QUIPS data base (see below) regardless of the anesthetic technique used, meeting the inclusion and exclusion criteria were evaluated. Inclusion criteria were as follows: (1) patients older than 18 years of age, (2) able to communicate and (3) who had primary unicompartmental or total knee replacement surgery performed. Information about QUIPS was supplied to all potential patients. Participation was voluntary. Informed consent was obtained from all participants. Exclusion criteria were: (1) absence of the patient in the ward, (2) patients who refused to participate and (3) patients who were asleep or sedated at the time of data collection and interviews 24 h postoperative.

The QUIPS questionnaire is divided into sections dealing with pain intensity, functional impairment, side effects of pain treatment and global assessment by the patient (compare Tables 3 and 5). Using the standardized QUIPS questionnaire form, minimum, maximum and activity pain were rated using the numeric rating scale “NRS” (NRS 0 = no pain, NRS 10 = worst pain imaginable). Furthermore, several side effects were monitored: “Vomiting after surgery”, “Felt nauseous after surgery”, “Felt vertiginous after surgery” and “Tired after surgery”. Additionally, functional outcome parameters “Pain affecting the ability to cough or to take a deep breath”, “Pain affecting the mood”, “Pain affecting the ability to sleep” and “Pain affecting the ability to move” were evaluated. The need for pain medication was obtained from patients’ records. The type of pain medication was classified according to the WHO pain ladder: non-opioids (WHO ladder step 1) and opioids (summarization of WHO ladder step 2 and step 3)^27^. For those patients, who needed an opioid postoperatively, the opioid equivalent was calculated in mg by using morphine as a basis for comparing the different opioid agonists.

All patients were randomly visited to avoid selection bias and patient-interviewer interaction bias. The surveyors were independent from the healthcare team. By using a standardized protocol to collect clinical data and obtain the questionnaire parameters, standardized data assessment was guaranteed. All collected data was anonymized.

### Statistical methods

**Figure 1:**
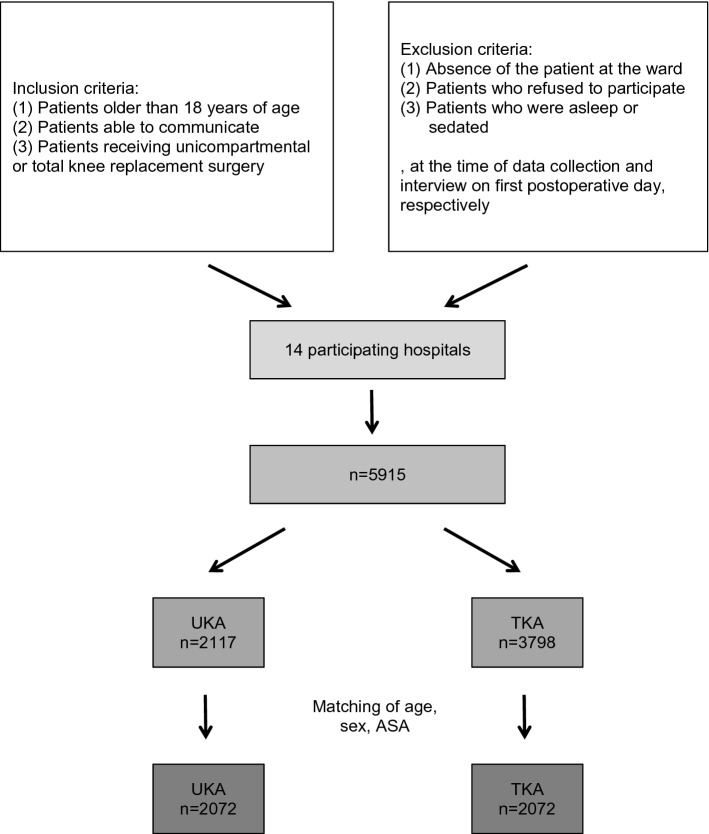
Flowchart: study group enrollment and matching.

Between 2015 and 2018, 5915 patients were included in the present cohort study after primary unicompartmental knee arthroplasty or total knee arthroplasty. The study was conducted nationwide in 14 orthopedic departments at the time of data evaluation. Patients were divided into two groups in question: UKA (n = 2117) and TKA (n = 3798). The following statistical evaluation was performed according to Greimel et al.^28,29^. To get comparable groups in size and distribution of the confounding variables a 1:1 match was performed. Patients of the UKA group (n = 2117) and the TKA group (n = 3798) were matched according to age, sex and ASA score. If there was more than one matching partner for one patient, one patient was randomly chosen. A total of 4144 patients were finally analyzed and compared (n = 2072, in each group, Fig. 1). After matching the age, sex and ASA score, both groups of UKA and TKA had comparable pain intensity preoperatively (Table 2).

Continuous variables were indicated by mean (standard deviation) or median (interquartile range) depending on the underlying distribution. Categorical data was presented as absolute numbers and/or relative frequencies. The opioid equivalent was calculated using the Mann–Whitney-U-test. To compare the use of pain medication, side effects or functional parameters between the UKA group and the TKA group, a Pearson’s chi-squared test was used for each pairwise comparison. The differences in the NRS values between the two study groups were analyzed by using t-tests. Normality was assessed visually by Q-Q-Plots and by the parameters median, mean, skewness and kurtosis. Normal distributed data were compared using students t-Test. Non-Normal data were compared using the Mann–Whitney-U Test. All reported *p*-values are two-sided and a *p *value < 0.05 was considered statistically significant. All analyses were performed using SPSS 25.0 (IBM SPSS Statistics, Armonk, NY—IBM Corp.).

## Results

A total of 4144 patients (n = 2072 per group, respectively) were finally statistically analyzed and compared after performing a matching of age, sex and ASA score because of demographic inhomogeneity and to reduce confounding variable bias (Fig. 1). In Tables 1 and 2 demographic and general data are shown before and after the matching. After matching, patients with UKA and TKA both showed a median pain of 6.0 (NRS) and mean pain of 6.4 (NRS) preoperatively (Table 2). Therefore, both groups of UKA and TKA had comparable pain intensity preoperatively. Furthermore, the choice of anesthesia before and after matching is shown in Tables 1 and 2.

**Table 1:** Demographic and general data before matching.

	TKA	UKA	ALL
Patients total (%)	3798 (64.2%)	2117 (35.8%)	5915 (100%)
Age in years (median, mean ± SD)	65, 67.6 ± 9.9	65, 65.3 ± 10.5	65, 66.7 ± 10.2
Sex in % (female:male)	61.1:38.9	55.4:44.6	59.1:40.9
ASA score [median, mean ± SD]	2, 2.3 ± 0.57	2, 2.17 ± 0.55	2, 2.26 ± 0.57
Operation time in minutes (median, mean ± SD)	70, 75 ± 28	78, 81 ± 36	72, 77 ± 31
Pain before surgery NRS (median, mean ± SD)	7.0, 6.5 ± 1.9	6.0, 6.4 ± 1.8	7.0, 6.5 ± 1.9
Anesthetic technique used (general : regional : combination) (%)	12.0 : 17.7 : 57.2	10.1 : 15.3 : 63.2	11.3 : 16.8 : 59.3

ASA—American Society of Anesthesiologists; UKA – unicompartmental knee arthroplasty, TKA – total knee arthroplasty; NRS—numeric rating scale; SD—standard deviation.

**Table 2:** Demographic and general data after matching.

	TKA	UKA	All
Patients total (%)	2072 (50%)	2072 (50%)	4144 (100%)
Age in years (median, mean ± SD)	65, 65.6 ± 10.1	65, 65.6 ± 10.1	65, 65.6 ± 10.1
Sex in % (female:male)	55.9 : 44.1	55.9 : 44.1	55.9 : 44.1
ASA score [median, mean ± SD]	2, 2.2 ± 0.5	2, 2.2 ± 0.5	2, 2.2 ± 0.5
Operation time in minutes (median, mean ± SD)	69, 75 ± 29	78, 81 ± 36	72, 78 ± 33
Pain before surgery NRS (median, mean ± SD)	6.0, 6.4 ± 1.9	6.0, 6.4 ± 1.8	6.0, 6.4 ± 1.8
Anesthetic technique used (general:regional:combination) (%)	11.2:18.7:63.9	10.1:15.3:63.2	10.7:17.0:63.5

ASA—American Society of Anesthesiologists; UKA—unicompartmental knee arthroplasty, TKA—total knee arthroplasty; NRS—numerous rating scale; SD—standard deviation.

**Figure 2:**
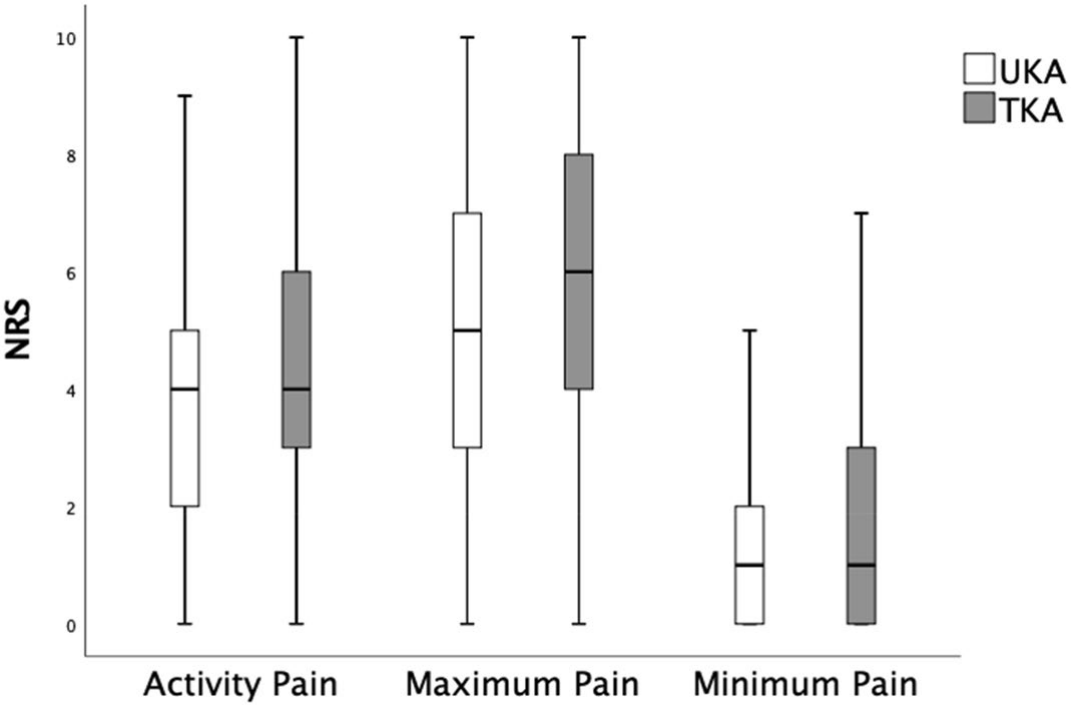
Bar charts: mean numeric rating scale (NRS) values and 95% confidence intervals for activity pain, maximum pain and minimum pain on the first postoperative day for patients with unicompartmental knee arthroplasty (UKA) and total knee arthroplasty (TKA).

In the UKA group mean activity pain was 4.1 (± 2.3) and in the TKA group 4.4 (± 2.4). Mean activity pain was significantly lower for the UKA group (*p* < 0.001) (Fig. 2, Table 3). Patients with UKA had a mean minimum pain of 1.6 (± 1.6) and mean maximum pain of 5.1 (± 2.6) whereas patients with TKA had a mean minimum pain of 1.8 (± 1.8) and a mean maximum pain of 5.6 (± 2.6). Mean maximum pain and mean minimum pain showed a statistically significant (each *p* < 0.001) advantage for the UKA group (Fig. 2, Table 3).

**Table 3:** Comparison of activity pain, maximum pain and minimum pain between “TKA” and “UKA” groups: mean values, standard deviation, and their significance levels.

	TKA	UKA	*p* values
Activity pain	4.4 ± 2.4	4.1 ± 2.3	< 0.001
Maximum pain	5.5 ± 2.6	5.1 ± 2.6	< 0.001
Minimum pain	1.8 ± 1.8	1.5 ± 1.6	< 0.001

UKA = unicompartmental knee arthroplasty, TKA = total knee arthroplasty, *p*-Values < 0.05.

In the recovery unit for UKA patients the need for non-opioids was significantly lower than for the TKA patients (*p* = 0.004). The opioid consumption was similarly lower for the UKA group, but not statistically significant (*p* = 0.15) (Table 4).

**Table 4:** Comparison of the need for pain medication until the first postoperative day between “UKA” and “TKA” groups and the opioid equivalent. PCA = patient controlled analgesia.

	TKA	UKA	*p-*Values
Recovery unit: Coanalgetics	8%	5%	0.011
Recovery unit: Nonopioids	50%	45%	0.004
Recovery unit: Opioids	57%	55%	0.152
Recovery unit: PCA	30.5%	32.9%	0.130
Ward: Coanalgetics	3.6%	4.2%	0.038
Ward: Nonopioids	94%	96%	< 0.001
Ward: Opioids	84%	76%	< 0.001
Ward: PCA	33.7%	34.5%	0.067
Recovery unit: Opioid equivalent (mg) (median, IQR 25%/75%)	0.0, 0.0/6.8	0.0, 0.0/7.5	0.35
Ward: Opioid equivalent (mg) (median, IQR 25%/75%)	15.0, 0.0/26.0	15.0, 0.0/30.0	0.27

In the ward, the percentage of patients who had taken opioids in the UKA group were statistically less than those in the TKA group (*p* < 0.001). The use for non-opioids was statistically higher for UKA than for TKA (*p* < 0.001). The calculation of the opioid equivalent (mg) in relation to morphine showed no statistically significant difference between the two groups (Table 4).

Functional outcome parameters and side effects are shown in Table 5. For the parameters “woke up because of pain”, ““felt nauseous after surgery”, “felt vertiginous after surgery”, “felt very tired after surgery” and “pain affected the mood”, significantly better results in the UKA group were demonstrated (*p* < 0.001 for all 5 items respectively). All other items did not differ significantly after comparing the two groups in question.

**Table 5:** Questions of the QUIPS-questionnaire. Functional outcome parameters (1) and side effects (2) on the first postoperative day.

	TKA	UKA	*p *values
Pain affecting the ability to sleep^1^	43%	35%	< 0.001
Pain affecting the ability to move^1^	71%	70%	0.991
Pain affecting the ability to cough or to take a deep breath^1^	3.0%	2.8%	0.78
Pain affecting the mood^1^	19%	13%	< 0.001
Tired after surgery^2^	39%	31%	< 0.001
Felt vertiginous after surgery^2^	24%	15%	< 0.001
Felt nauseous after surgery^2^	21%	15%	< 0.001

## Discussion

In recent years many studies have been carried out on the comparison of the clinical outcomes of patients after UKA and TKA. Among these studies, the consensus conclusion, is that patients who underwent UKA have better function PROM scores, better range of movement, quicker recovery period and shorter hospitalization^15,16,30^, but no difference in pain, comparing UKA to TKA^13,15,16^. These studies in general used PROM scores (KSS/WOMAC) for evaluation of pain intensity in the follow-up after UKA and/or TKA. Literature evaluating early postoperative pain and pain management after UKA and TKA is emerging. In addition, possible side effects of pain therapy and functional impairments were rarely investigated. The aim of this study was to compare the use of pain medication, pain control and patient’s subjective parameters after having had UKA or TKA performed as early pain control can have an impact on postoperative outcome and length of hospitalization.

Similar results to our study can be seen in the study of Melnic et al.^31^ in which 71 patients with UKA and 37 patients with PFA (patellofemoral arthroplasty) were matched by sex and age to 108 patients with TKA. Opioid consumption in the first postoperative ward round was significantly lower for the UKA group than for the TKA group or PFA. A consistent result was found in the Kalbian et al. study^32^. Patients required a significantly lower rate of opioid prescription after UKA-implantation compared to TKA-implantation. We considered that the reduced consumption of opioids after UKA implantation was due to lesser surgical trauma caused by a smaller incision and a greater perseveration of native structures.

We did not expect anesthesia procedures to represent a potential confounder, as anesthesia procedures did not differ in percentage between the two groups of UKA and TKA (Table 2).

For those patients who needed an opioid, the calculation of the opioid equivalent in the recovery unit and in the ward showed no significant difference between the two groups. One possible explanation could be an increased individual need for opioids. In total, patients after UKA had a lower opioid consumption compared to patients after TKA, but those who had taken an opioid may have needed a comparatively higher dose, since significantly fewer patients required an opioid after UKA than after TKA by an equivalent opioid dose (Table 4). On the other hand, our data showed a significantly higher percentage of non-opioid use within the UKA group at the ward, which might have had an impact on the opioid consumption as well.

Another important parameter was the patient’s subjective pain level. A lower postoperative pain level means increased comfort and satisfaction for the patient and consequently an improved postoperative function. Postoperatively, there was a significant difference in pain under mobilization, measured according to the NRS-scale, with less pain in the UKA group in comparison to the TKA group. The pain maximum and pain minimum (NRS) were also significantly lower for the UKA group. (Fig. 2, Table 3).

To date, there are hardly any studies that have investigated early postoperative pain differences between UKA and TKA implantation. In general, pain was investigated using PROM scores, e.g. the KS pain score or the WOMAC score. In the systematic review and meta-analysis of Wilson et al. ^15^ pain specific PROM scores were found to be equivocal after UKA compared with TKA with no significant difference between the two groups. The matched pair analysis of Lombardi et al. ^13^ showed no significant difference in pain after UKA and TKA in the KS pain score after a follow up of 6 weeks and an average of 30 months. The study by Noticewala et al. reported a significant difference in the WOMAC pain score and the WOMAC physical function score in the follow-up 3 years after UKA or TKA with better result for UKA^33^. A recently published study by Lakra et al.^34^ has shown that an improved management of early postoperative pain was associated with a better functional outcome in the follow-up 2-years after TKA. Our results showed lower pain under mobilization and lower minimum and maximum pain for the UKA group. Higher postoperative patient-reported functional outcomes were described in several studies with advantages for UKA^6,8,14,15,26^.

The risk of developing chronic pain after knee surgery is reported to be even higher than in e.g. hip replacement surgery^35^. A correlation between the postoperative pain level and the development of chronic pain has been shown^22,23^. An improvement in pain management in the early postoperative course could be a contributing factor in reducing chronic pain. This could lead to faster recovery and mobilization, psychological well-being and to a general improvement of the outcome.

Furthermore, the group of UKA had significantly better results for side effects “very tired”, “vertiginous”, “nausea” and functional outcome parameters: “woke up because of pain” and “pain affecting mood” (Table 5). The patient's quality of life and early postoperative mobilization can lead to functional improvements. In the recovery unit, there was no statistically significant difference in opioid consumption between the UKA and TKA group, but the parameters vertiginous and nausea could be influenced by opioid consumption.

This study shows several limitations such as the assessment of postoperative pain and pain management. Because of organizational reasons these values have been collected on the first postoperative day and differences in the continuing postoperative period could not be evaluated. Within the QUIPS study protocol, factors such as indication criteria for UKA or TKA, intraoperative surgical details such as the use of tourniquets or drains could not be investigated.

Big data studies have a general restriction in explanatory power. A huge data quantity could provide significant results despite small differences in value. Nevertheless, in daily clinical routine big data studies are of great interest to evaluate the efficacy of interventions.

Furthermore, selection bias cannot be barred, but the matching tries to reduce the influence of confounding variables. One possible selection bias could have been preoperative pain intensity and anesthetic technique used, but after matching both groups showed similar pain intensity preoperatively. We have demonstrated statistically significant results for pain level between the both groups of UKA and TKA, but possibly they are below clinical relevance. This big data study represents daily clinical routine and is therefore, of great interest in the clinical decision-making process in choosing treatment options. A high percentage of patients, who are eligible for primary joint replacement with isolated unicompartmental osteoarthritis still have TKA performed.

## Conclusions

In contrast to previous studies, our study has shown that patients undergoing UKA have had lower pain scores postoperatively and less need for opioids in the ward, in a multicenter matched pair analysis in 4144 cases. Furthermore, patient subjective parameters were significantly better for UKA. Although opiate consumption was less likely for patients with UKA than for patients with TKA, there was no significant difference in opiate equivalent.
